# Origins of Parkinson’s Disease in Brain Development: Insights From Early and Persistent Effects of LRRK2-G2019S on Striatal Circuits

**DOI:** 10.3389/fnins.2020.00265

**Published:** 2020-03-26

**Authors:** George W. Huntley, Deanna L. Benson

**Affiliations:** Nash Family Department of Neuroscience, Friedman Brain Institute, Graduate School of Biomedical Sciences, Icahn School of Medicine at Mount Sinai, New York, NY, United States

**Keywords:** LTP, corticostriatal, development, social defeat stress, striatum, nucleus accumbens, intrinsic excitability

## Abstract

Late-onset Parkinson’s disease (PD) is dominated clinically and experimentally by a focus on dopamine neuron degeneration and ensuing motor system abnormalities. There are, additionally, a number of non-motor symptoms – including cognitive and psychiatric – that can appear much earlier in the course of the disease and also significantly impair quality of life. The neurobiology of such cognitive and psychiatric non-motor symptoms is poorly understood. The recognition of genetic forms of late-onset PD, which are clinically similar to idiopathic forms in both motor and non-motor symptoms, raises the perspective that brain cells and circuits – and the behaviors they support – differ in significant ways from normal by virtue of the fact that these mutations are carried throughout life, including especially early developmental critical periods where circuit structure and function is particularly susceptible to the influence of experience-dependent activity. In this focused review, we support this central thesis by highlighting studies of LRRK2-G2019S mouse models. We describe work that shows that in G2019S mutants, corticostriatal activity and plasticity are abnormal by P21, the end of a period of excitatory synaptogenesis in striatum. Moreover, by young adulthood, impaired striatal synaptic and non-synaptic forms of plasticity likely underlie altered and variable performance by mutant mice in validated tasks that test for depression-like and anhedonia-like behaviors. Mechanistically, deficits in cellular, synaptic and behavioral plasticity may be unified by mutation-linked defects in trafficking of AMPAR subunits and other membrane channels, which in turn may reflect impairment in the function of the Rab family of GTPases, a major target of LRRK2 phosphorylation. These findings underscore the need to better understand how PD-related mutant proteins influence brain structure and function during an extended period of brain development, and offer new clues for future therapeutic strategies to target non-motor cognitive or psychiatric symptoms of PD.

## Introduction

Late-onset Parkinson’s disease (PD) is a movement disorder diagnosed clinically by the appearance in middle age of progressively debilitating motor symptoms, including rigidity, resting tremor, bradykinesia, postural instability, and gait disturbances, among others (Parkinson, 2002). Such primary motor disturbances result principally from progressive death of dopamine (DA) neurons in the substantia nigra and accompanying degenerative loss of DA axon terminals within striatum. Treatment of motor symptoms accordingly relies on DA replacement strategies, which become less effective over time and ultimately produce dyskinesia (Bastide et al., 2015).

Less well understood, both clinically and mechanistically, is a spectrum of prominent non-motor symptoms that appear during a temporally variable prodromal phase occurring prior to onset of the disease-defining motor symptoms (Schapira et al., 2017). Such non-motor symptoms include loss of sense of smell, sleep disturbances, gastrointestinal problems, and other forms of autonomic dysfunction (Savica et al., 2010). Additionally, cognitive decline (deficits in working memory, cognitive flexibility, attention, and reinforcement learning) and psychiatric symptoms (depression, anxiety) are common, greatly diminish quality of life and, for many of these symptoms, can also appear during the early prodromal phase of the disease (Grover et al., 2015). In some cases, it is thought that depression and anxiety, prior to motor symptoms, may be causal risk factors for PD (Lin et al., 2014; Gustafsson et al., 2015). The underlying neurobiology of cognitive and psychiatric symptoms of PD is not well understood. However, the onset of cognitive non-motor symptoms may be largely independent of overt DA neuron degeneration since their early appearance likely antedates significant DA neuron loss (Savica et al., 2010; Volta et al., 2015; Sloan et al., 2016) and DA agonists used to treat motor symptoms are weakly effective anti-depressants (Starkstein and Brockman, 2017). It is possible that other modulatory systems, for example, serotonergic systems, may be involved (Mayeux et al., 1984).

The clinical management of PD largely focuses on the late-onset motor symptoms, leading in some cases to an almost tacit view that the cellular and synaptic environment in brains of PD patients is normal until the prodromal stage, at which time some pathophysiological process arises to co-opt and disrupt brain circuits and set a course of steady, degenerative decline. Based in part on genetic forms of late-onset PD and the mouse models used to mechanistically interrogate the impact of such mutations on cell and circuit function, there is growing recognition that this view probably does not adequately capture the complexity of the disease process or the cellular/circuit environment in the brain in which the disease manifests (Hemmerle et al., 2012; Irwin et al., 2013; Kannarkat et al., 2013; Benson and Huntley, 2019). In this focused review, we highlight mouse studies of LRRK2 and the prevalent G2019S mutation to underscore the broader, central thesis that PD-related gene mutations – present during brain development and beyond – exert significant effects on establishment and maturation of relevant circuits that impact their function, and perhaps viability, throughout life.

## Why Study the Effect of a PD Gene Early in Life?

LRRK2 is a large, multifunctional protein in which the G2019S gain-of-kinase activity point mutation is the most prevalent cause of autosomal dominant, heritable forms of late-onset PD. Further details on LRRK2 structure, targets and general biology can be found in other articles in this collection. The clinical presentation of LRRK-G2019S carriers, including both motor and non-motor symptoms, is similar to idiopathic PD, and the majority of less common pathological LRRK2 mutations appear to act through mechanisms similar to G2019S (Greggio et al., 2006; Smith et al., 2006; Paisan-Ruiz et al., 2013; Mir et al., 2018). While a variety of LRRK2 mouse models have been described, it is important to clarify at the outset that none of these should be considered faithful models of PD *per se* – that is, displaying all of the cardinal features of the disease process (Dawson et al., 2010; Blesa and Przedborski, 2014). Rather, they provide mechanistic insight into the role of LRRK2 in cell and circuit function, and how the G2019S mutation can derail, modulate or otherwise influence cells, circuits and ultimately behaviors, at all stages of life.

**FIGURE 1:**
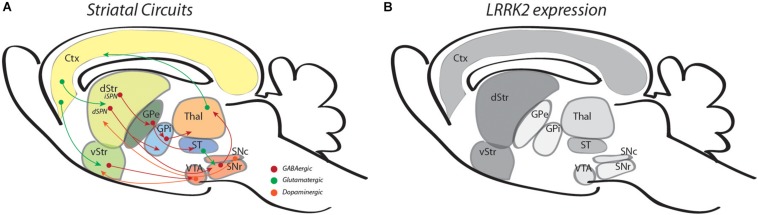
Illustrations outline striatal circuits **(A)** and relative levels of LRRK2 expression in the same regions **(B)**. In panel **A**, green arrows are used for glutamatergic circuits, red, for GABAergic and orange, for dopaminergic circuits. In panel **B**, different shades of gray are used to represent the approximate intensity of LRRK2 mRNA levels observed (references in text). Ctx, cortex; d/vStr; dorsal and ventral striatum; GPe/i, globus pallidus external and internal; Thal, thalamus; ST, subthalamus; VTA, ventral tegmental area, SNr/c, substantia nigra pars reticulata, compacta.

The normal function of LRRK2 in brain is not completely understood. One starting point to infer function is to consider where and when LRRK2 is expressed. In mice or humans, LRRK2 expression in brain is particularly enriched in dorsal and ventral striatum and cerebral cortex but is only weakly expressed in DA neurons of the substantia nigra or ventral tegmental area (Figure 1; Giesert et al., 2013; West et al., 2014; Sandor et al., 2017). In mouse striatum, single-cell RNA sequencing has shown that LRRK2 expression levels are high in both direct (D_1_R)-pathway SPNs (dSPNs) and indirect (D_2_R)-pathway SPNs (iSPNs) (Figure 1), are somewhat lower in a variety of interneurons and astrocytes, and even lower in (non-activated) microglia (Gokce et al., 2016). Developmental anatomical and biochemical studies in rodents demonstrate that levels of LRRK2 expression in striatum are low at birth, but rise significantly during the first three postnatal weeks (through P21) and remain elevated into adulthood (Westerlund et al., 2008; Giesert et al., 2013). This early postnatal period of rising LRRK2 expression levels in striatum is significant for two principal reasons: first, it is contemporaneous with the ingrowth of corticostriatal afferents and a rapid burst in excitatory synaptogenesis (Sharpe and Tepper, 1998; Tepper et al., 1998; Sohur et al., 2014); and second, this early postnatal period corresponds to a developmental “critical period” where establishment of structural and functional features of synaptic connectivity show heightened sensitivity to changing levels, patterns or timing of neural activity, particularly that driven by experience (Kozorovitskiy et al., 2012; Greenhill et al., 2015; Molero et al., 2016; Peixoto et al., 2016). This is illustrated by an experiment in which L5 corticostriatal neuron activity was chemogenetically inhibited transiently during the second week of postnatal development, then returned to normal levels. Immediately following this period of neural activity silencing, mEPSC frequency and dendritic spine density were decreased in both dSPNs and iSPNs, but these changes persisted into young adulthood despite restoration of neural activity (Kozorovitskiy et al., 2012). Relationships between early experience or exposure can be complex and may not be immediately evident. For example, when mutant Huntingtin is expressed transiently in mice until P21, striatal neurons display functional abnormalities and degenerative phenotypes at 9 months of age, similar to what is observed in mice constitutively expressing mutant Huntingtin (Molero et al., 2016). Interestingly, many of the consequences are not evident at 3 months of age and support the idea that pathology can emerge long after exposure (Molero et al., 2016). These observations provide a framework for two interrelated ideas: the first is that LRRK2 and its pathogenic mutations are positioned to affect development of corticostriatal circuits; and second, an early influence on circuit development will have persistent or newly emergent consequences for altered or compensatory function throughout life. Consistent with these ideas, functional imaging studies of human non-manifesting carriers (NMCs) of the LRRK2-G2019S mutation or non-manifesting non-carrier (NMNC) controls have shown differences between groups in functional network activity, changes that may underlie early alterations in executive function and reward-based neural processing. For example, in NMCs, fMRI studies have shown abnormalities in corticostriatal circuit organization in comparison with controls (Helmich et al., 2015) and changes in the resting-state non-motor-related default networks that precede later changes in the resting state motor-related network (Jacob et al., 2019). These differences may underlie diminished executive function (Thaler et al., 2012) and different, perhaps compensatory patterns of task-related activity during certain cognitive tests displayed by NMCs (Thaler et al., 2013). Additionally, in comparison with controls, NMCs display disturbances in reward processing and abnormal neural activity in ventral striatum (Thaler et al., 2019).

## LRRK2 Mutation Regulates Glutamatergic Activity During Development

LRRK2-G2019S knock-in mice, in which the mutant protein is expressed at levels similar to wildtype LRRK2, have been particularly valuable for examining the impact of mutation on developing striatal circuits. Whole-cell recordings from dorsomedial SPNs in acute wildtype or G2019S slices have shown a large increase (about 4-fold) in frequency of spontaneous excitatory postsynaptic currents (sEPSCs) in both dSPNs and iSPNs at P21, the height of corticostriatal synaptogenesis. Similar results were observed at P28 in dorsolateral striatum (Volta et al., 2017). The magnitude of the abnormality in sEPSC frequency is equivalent in G2019S heterozygous and homozygous mice, consistent with the autosomal dominance of the mutation in humans (Paisan-Ruiz et al., 2004; Zimprich et al., 2004), and between male and female mice. Such abnormal activity appears to originate presynaptically from corticostriatal axons based on three observations: first, acute, surgical separation of the striatum from the overlying cortex in mutant slices restores sEPSC frequency to wildtype levels (Matikainen-Ankney et al., 2016); second, cultured cortical neurons show increased EPSC frequency (Beccano-Kelly et al., 2014a); and third, there are no differences between wildtype and G2019S mutants in baseline intrinsic excitability of SPNs (Matikainen-Ankney et al., 2016; Volta et al., 2017). Intriguingly, in our studies, abnormally heightened activity in the G2019S SPNs is of the kind that depends on action potentials (APs) because following bath-application of TTX (which blocks generation of APs), there is only a small, statistically insignificant effect on frequency of mini-EPSCs (mEPSCs) (Matikainen-Ankney et al., 2016). In contrast, studies of cortical neurons cultured from G2019S knockin mice and recorded in the presence of TTX found a significant increase in mEPSC frequency compared to wildtype cortical neurons (Beccano-Kelly et al., 2014a), suggesting that at mutant corticocortical synapses, AP-independent events may be a major contributor to heightened activity. It is important to keep in mind that spontaneously occurring EPSCs – either AP-dependent or AP-independent – represent a cumulative mixture of potentially different convergent inputs, each of which may be differentially affected by the mutation in a synapse-, cell-, region- or age-dependent fashion (Sweet et al., 2015; Yue et al., 2015; Pan et al., 2017). In striatum, for example, EPSCs could originate from cerebral cortex, thalamus, amygdala, hippocampus, or other sources all converging onto single SPNs from which EPSCs are recorded. In any event, the seemingly selective effect of the G2019S mutation on AP-dependent activity when recording EPSCs in dorsal striatum, as opposed to stochastic, AP-independent release of vesicles, may be a potentially important distinction. While it is generally accepted that LRRK2 plays a role in synaptic vesicle recycling (Shin et al., 2008; Piccoli et al., 2011; Matta et al., 2012; Parisiadou et al., 2014; Arranz et al., 2015; Belluzzi et al., 2016; Pan et al., 2017), TTX-sensitive and -insensitive vesicle release can involve different pools of neurotransmitter-containing synaptic vesicles and molecular pathways (Chanaday and Kavalali, 2018), potentially offering clues to the identity of molecular substrates of LRRK2-G2019S important for altering synaptic activity levels. At the same time, as discussed above, there may be synapse or region specific effects (Sweet et al., 2015; Pan et al., 2017) that will have to be taken into consideration. Convergent genetic and pharmacological approaches demonstrate that the abnormally elevated activity in G2019S mutant slices depends on the elevated kinase activity of the mutation. Whole-cell recordings from SPNs in acute striatal slices from a LRRK2-D2017A kinase-dead mutant or LRRK2 kinase inhibitors bath-applied to G2019S striatal slices both reduce the abnormally elevated activity to wildtype levels (Matikainen-Ankney et al., 2016). Importantly, neither genetic ablation of LRRK2 kinase activity nor pharmacological inhibition of LRRK2 kinase activity in wildtype slices lowers sEPSC frequency to levels below wildtype values (Matikainen-Ankney et al., 2016), consistent with the absence of an effect of LRRK2 knockout on sEPSC frequency (Beccano-Kelly et al., 2014b). These outcomes indicate that LRRK2 kinase activity *per se* is not normally required for glutamatergic vesicle release at these synapses. Rather, the elevated AP-dependent increase in sEPSC frequency in G2019S slices most likely represents a gain-of-abnormal function imparted by the mutation, although it remains possible this is an indirect, compensatory effect of the mutation. Other domains of LRRK2 may function in neurotransmitter vesicle release at earlier ages, since mEPSC frequency recorded from SPNs in P15 LRRK2 knockout mice are lower than wildtype (Parisiadou et al., 2014). The elevated frequency of sEPSCs evident by P21 does not reflect an increase in synapse density, and it is developmentally transient, returning to wildtype levels by young adulthood (Matikainen-Ankney et al., 2016; Volta et al., 2017; Tozzi et al., 2018).

In addition to increased sEPSC frequency – likely presynaptic in origin as discussed above – postsynaptic effects in G2019S dorsomedial SPNs are also evident by P21. Cumulative probability distributions show that dendritic spine-heads are larger in comparison with those on wildtype SPNs. Since generally larger spines are correlated with larger AMPAR currents (Matsuzaki et al., 2001), predictably the larger spines on mutant SPNs are matched by larger sEPSC amplitudes in comparison with those recorded from wildtype SPNs (Matikainen-Ankney et al., 2016). It is not clear whether the spine-head size and current amplitude effects described for dorsomedial SPNs are a direct result of the G2019S mutation within SPNs, or an indirect effect resulting from the excessive corticostriatal activity during this period. However, in ventral striatal SPNs from the same line of knockin mice at the same age (P21), a similar enlargement of spine-head sizes and current amplitudes is evident but sEPSC frequency is unchanged in comparison with wildtype (Guevara et al., 2020), indicating that in this population of SPNs, postsynaptic effects on spine-head size and amplitude cannot be attributable to an indirect consequence of an elevation in presynaptic activity. This underscores an important point that bears emphasis – cellular and synaptic effects of G2019S (or any other PD-related mutation) does not necessarily manifest identically across the cells and circuits in which the mutant protein is found (Sweet et al., 2015; Pan et al., 2017). It is not known if such morphological changes in spine-head sizes evident at P21 persist into adulthood.

## Impact of LRRK2 Mutation on Synapse Plasticity Over the Lifespan

In addition to alterations in spontaneously elicited baseline currents (EPSC frequency and amplitude), corticostriatal synapses on dorsomedial G2019S SPNs also exhibit aberrant evoked responses – namely, mutant SPNs are unable to express bidirectional synaptic plasticity (Matikainen-Ankney et al., 2018). Bidirectional synaptic plasticity is the ability of synapses to undergo activity-dependent long-term potentiation (LTP), a persistent increase in synaptic strength, or long-term depression (LTD), a persistent decrease in synaptic strength. In striatum, LTP is NMDAR-dependent and postsynaptically mediated (Kreitzer and Malenka, 2008; Ma et al., 2018), while LTD is presynaptically mediated by eCB1 receptor activation on glutamatergic terminals which reduces probability of neurotransmitter release (Calabresi et al., 1992; Choi and Lovinger, 1997; Kreitzer and Malenka, 2007). Current models indicate that both dSPNs and iSPNs can undergo LTP and LTD (Kreitzer and Malenka, 2007; Shen et al., 2008; Higley and Sabatini, 2010), with the direction of synaptic plasticity controlled by opponent mechanisms involving GPCR (Gs or Gi) signaling cascades. Experimentally disrupting such signaling cascades by 6-OHDA or other chemical lesions does not prevent striatal synaptic plasticity, but renders it abnormally unidirectional (Picconi et al., 2003; Kreitzer and Malenka, 2007; Shen et al., 2008). This is potentially significant, because striatally based learning in PD patients is dysfunctional rather than completely absent (Dujardin et al., 2003). In G2019S mice, dSPNs and iSPNs in dorsomedial striatum are unable to express LTP, an impairment that is present by P21 and persists into adulthood (Matikainen-Ankney et al., 2018). In fact, in G2019S SPNs, a pairing-stimulus protocol that normally leads to LTP in wildtype SPNs produces instead an abnormal LTD, which is most pronounced for iSPNs. This may be due in part to abnormal DA levels and/or enhanced sensitivity of D_2_R signaling. Repeated stimulation in striatum of G2019S slices produces significantly greater peak levels of DA release and longer DA decay times than wildtype (Volta et al., 2017) and sEPSCs in G2019S SPNs, but not wildtype SPNs, are reduced by D_2_R activation via a retrograde, CB1-receptor dependent signaling pathway that would be anticipated to enhance LTD (Tozzi et al., 2018). Interestingly, this latter effect is not reversed by pharmacological inhibition of LRRK2 kinase activity, suggesting it may reflect developmentally imposed changes in wiring (Tozzi et al., 2018). Finally, other G2019S mouse models have shown deficits in high-frequency stimulation-induced LTD in aged striatum (Chou et al., 2014), and an age-related loss of LTD in hippocampus (Sweet et al., 2015). While these observations reinforce the idea that effects of the mutation are likely age, cell and synapse specific, it is prudent to also consider that some of these effects may be attributable to cellular and regional idiosyncracies in the expression levels or patterns of wildtype or G2019S LRRK2 in the different mouse models (e.g., knockin versus BAC transgenic overexpression).

## G2019S Mutation Alters Striatal-Dependent Behaviors

Together, these data raise the question of whether such early changes in striatal circuit structure/function coupled with early and persistent loss of bidirectional striatal synaptic plasticity in G2019S mice would have a lasting impact on striatally based behaviors. Several studies, utilizing different lines of G2019S knockin mice, have reported modest motor-like effects mostly appearing between 3 and 6 months of age or older (Volta and Melrose, 2017). Generally, such studies used only male mice. In one line of knockin mice, some hyperactivity in homozygous (but not heterozygous) animals was observed, which may indicate a gene dose-dependent effect of the mutation (Longo et al., 2014). In another line of knockin mice, enhanced exploratory activity and cylinder rearing was observed (Longo et al., 2014; Yue et al., 2015; Volta et al., 2017). Other studies using a third line of G2019S knockin mice found no differences with wildtype mice in motor-skill acquisition as assessed by performance on an accelerating rotorod nor in open-arena exploration (Matikainen-Ankney et al., 2018). Striatal circuits are also critical for goal-directed learning, action-outcome selection and habits (Balleine et al., 2007; Shiflett et al., 2010; Smith and Graybiel, 2014) but the effects of the mutation on these behaviors in mouse models is unknown. Further, ventral striatal circuits, important for reward, motivation and other behaviors, have been implicated in the pathophysiology of depression and anhedonia (Carlezon et al., 2005; Bosch-Bouju et al., 2016; Han and Nestler, 2017). In rodents, depression-like and anhedonia-like behaviors requiring plasticity in ventral striatal circuits can be tested by a variety of validated tests, including social defeat stress, sucrose-preference, and self-grooming. Social defeat is a paradigm where an experimental mouse (in our case, a wildtype or G2019S mouse, both of which are on a C57BL/6N background) is subjected to brief periods (5 min) of daily physical subordination by a large, aggressive CD1 retired male breeder. In all other time between bouts of physical interaction, defeated mice and their subordinator are housed together, but separated by a perforated plexiglass divider, allowing sensory, but not physical, contact. Following social defeat, mice are given a social interaction test in which they are allowed to explore an arena in the absence and subsequent presence of a novel social target that is constrained by a wire mesh enclosure at one end of the arena. Many studies have established that in a typical cohort of wildtype mice that undergo 10 days of social defeat undergo 10 days of social defeat stress (10-day-SDS), all defeated mice exhibit equal exploratory behavior in the arena in the absence of the social target, but in the subsequent presence of the social target, about half are socially curious and interactive, preferring to spend more time exploring the vicinity of the novel social target rather than other parts of the arena (so-called “resilient” mice), while the other half are significantly socially avoidant, preferring to spend more time in the far corners of the arena (Golden et al., 2011). The mice that display social avoidance are described as displaying a depression-like phenotype, since these animals typically also show anhedonia-like behaviors (tested by sucrose preference or self-grooming) and undergo a reversal in such behaviors when chronically administered anti-depressants (Berton et al., 2007).

In behaviorally naive young adult (2–3 month-old) male wildtype and G2019S mice, the mutation alone, in the absence of any particular prior experience, is generally insufficient for altering motor coordination, anxiety, exploratory activity, self-care and anhedonia-like behaviors (Volta and Melrose, 2017; Matikainen-Ankney et al., 2018). Additionally, behaviorally naive G2019S mice exhibit social interaction behavior that is indistinguishable from wildtype mice (Matikainen-Ankney et al., 2018). When multiple, independent cohorts of young adult male G2019S and wildtype mice were subjected to 10-day-SDS followed by a social interaction test, wildtype cohorts yielded expected ratios of socially interactive (57%) and socially avoidant (43%) subpopulations, while in contrast, G2019S mice were essentially all highly (and surprisingly) socially interactive (∼94%) despite 10-days of defeat experience (Matikainen-Ankney et al., 2018). Additionally, such “resilient” G2019S mice exhibited less anhedonia-like behaviors compared to defeated wildtype mice (that is, they drank more sucrose in a sucrose preference test and spent more time self-grooming in a sucrose-splash test).

One interpretation of this behavioral outcome is that this is a type of learning deficit. However, studies testing the social interaction behavior of G2019S mice after only 1-day of social defeat stress (1-day-SDS) complicates this interpretation. G2019S mice subjected to 1-day-SDS are all significantly more socially avoidant compared to 1-day-SDS wildtype mice (Guevara et al., 2020), another surprising outcome given the predominant resilience of G2019S mice to 10-day-SDS described above (Matikainen-Ankney et al., 2018). Additionally, after 1-day-SDS, socially avoidant G2019S mice drink significantly more sucrose (display less anhedonic-like behavior) than 1-day-SDS wildtype mice. Thus, in this case, the mutant mice display an unexpected “uncoupling” of social avoidance behavior and hedonic-like behavior. The neural basis for such behavioral differences between genotypes may lie in very different modes of adaptive plasticity in response to acute stress. In 1-day-SDS wildtype mice, SPNs in NAc display an adaptive, stress-induced increase in intrinsic excitability that is completely lacking in 1-day-SDS mutant SPNs. Instead, SPNs from 1-day-SDS G2019S mice show stress-induced changes in synaptic properties (increases in both sEPSC frequency and amplitude) that wildtype neurons lack (Guevara et al., 2020). Together, these behavioral and cellular and synaptic outcomes suggest a few things. First, while it is unclear at the moment what the significance of the altered and variable social behaviors in G2019S mice is, they appear not to conform to simple “depression-like” or “resilient-like” categorization; second, the amount and type of stress is likely to be critically important for driving a temporally evolving set of adaptive cellular, circuit, and/or synaptic changes that remain largely undefined at this point but which may vary significantly from wildtype mice. Additionally, the effect of age on these behaviors is unknown. It is possible that as these G2019S knockin mice age, more consistent depression- and anxiety-like behaviors would predominate, as suggested by studies of aged transgenic G2019S overexpressing mice (Lim et al., 2018).

## LRRK2, Plasticity, and Behavior

This leads us to ponder a final question: what are the underlying mechanisms that unify both the cellular/synaptic plasticity deficits and altered behavioral outcomes? The answer, though incomplete at this point, will likely include deficits in trafficking of AMPARs and other relevant membrane channels. Using combinations of electrophysiology and pharmacology applied to acute wildtype or G2019S slices from young adult mice, the data support that at baseline, evoked AMPAR-mediated currents at glutamatergic synapses in ventral striatum are mediated by a different composition of AMPAR subunits, with fewer in the mutants that are calcium permeable (CP) in comparison with wildtype. This is potentially significant, because dynamic trafficking of CP-AMPARs, such as GluA1, into the synaptic membrane is tied mechanistically to both LTP (Plant et al., 2006; Ma et al., 2018; Zhou et al., 2018) and the expression of social avoidance in mice undergoing 10-day social defeat (Vialou et al., 2010). Thus, it is reasonable to expect that baseline differences between genotypes in AMPAR stoichiometry would predict both the defects in lasting forms of synaptic plasticity and behavioral outcomes that depend on such plasticity. Following 10-day-SDS ventral striatal glutamatergic synapses in mice that are socially avoidant acquire enhanced sensitivity to NASPM, an antagonist of CP-AMPARs, and display inwardly rectifying current–voltage relationships at positive membrane potentials (Vialou et al., 2010; Matikainen-Ankney et al., 2018), both of which are signatures of incorporation of CP-AMPARs (Hume et al., 1991; Verdoorn et al., 1991). “Resilient” 10-day-defeated G2019S mice all retain linear AMPAR current–voltage relationships, similar to “resilient” wildtype mice, consistent with domination by non-CP-AMPAR subunits, such as GluA2. Since it has been posited that synaptic incorporation of CP-AMPARs promotes, at least in part, subsequent social avoidance behavior (Vialou et al., 2010), the lack of CP-AMPAR responses in G2019S mice could reflect an inability to traffic GluA1 or other CP-AMPARs into the membrane, rendering mice “resilient” to 10-day-SDS. If the foregoing is true, then the pronounced social avoidance of the G2019S mice observed after 1-day-SDS must reflect other mechanisms such as changes in intrinsic excitability (Francis et al., 2015; Guevara et al., 2020). It is also possible, but at present unexplored, that such adaptive changes in circuits or behaviors are driven in part by effects of the mutation that extend beyond the nervous system *per se* to include G2019S effects on the peripheral immune system, where LRRK2 is enriched, resulting in aberrant modulatory effects on brain cells and circuits by immune cells (Dzamko, 2017). It has been shown, for example, that in mice that eventually display social avoidance or anxiety following social defeat stress, peripheral myeloid cells and cytokines gain access to the brain and can influence social interaction following social defeat (Wohleb et al., 2011; Hodes et al., 2014; Yin et al., 2019).

A link between the G2019S mutation and hypothesized deficits in membrane channel trafficking is provided by considering the Rab family of GTPases as a significant target of LRRK2 phosphorylation (Steger et al., 2016, 2017) within the Rab effector-binding motif (Stroupe and Brunger, 2000). Rab8, an established phospho-target of LRRK2, regulates AMPAR insertion into synapses in hippocampal neurons and could be playing a similar role in SPNs (Brown et al., 2007). That trafficking pathways are relevant gains support from data showing that Rab7L1/Rab29, a PD risk factor gene, activates LRRK2, promotes its location to Golgi, and potentiates its kinase activity (Kuwahara et al., 2016; Liu et al., 2018; Purlyte et al., 2018). PD mutations in VPS35, a part of the retromer complex, also serve to potentiate the actions of LRRK2 kinase activity (Mir et al., 2018) and impact AMPA receptor recycling in cortical and hippocampal neurons and hippocampal LTP (Munsie et al., 2015; Temkin et al., 2017). It is also possible that PKA pathways contribute. PKA signaling is altered in the absence of LRRK2 (Parisiadou et al., 2014) and a recent paper suggests that LRRK2 can regulate phosphodiesterases, which regulate cAMP degradation upstream of PKA (Russo et al., 2018). Finally, it is possible that G2019S contributes to altered development and function of striatal or other synaptic circuits in part through mechanisms involving aberrant protein synthesis (Martin et al., 2014a). LRRK2 putatively interacts with and phosphorylates several ribosomal proteins (reviewed in Taymans et al., 2015), and previous studies of flies or using human induced pluripotent stem cells have shown that LRRK enhances both cap-dependent and -independent translation (Martin et al., 2014b; Penney et al., 2016). This is potentially relevant to the behavioral and synaptic abnormalities resulting from a putative developmental effect of the G2019S mutation because in a mouse model of autism, enhancing cap-dependent translation by increasing levels of the translation initiation factor eIF4E drives early synaptic plasticity deficits in striatum and elsewhere and produces aberrant striatal-dependent cognitive and social behaviors (Santini et al., 2013) similar to what has been described for Fragile X Syndrome (Bear et al., 2004; Sorensen et al., 2015). Thus, G2019S-mediated alterations in protein synthesis could be a contributing factor to the loss of LTP and aberrant stress-induced social interaction behaviors described above.

Collectively the data support that LRRK2 mutation alters vesicle recycling, trafficking and possibly protein synthesis during development in ways that are sustained, impacting certain cellular behaviors over the lifespan, and in other ways that are transient, but have permanent consequences for the assembly of neural circuits and the eventual behaviors they support. Given the impact of the G2019S mutation on LTP in the dorsal striatum, it is likely that striatal behaviors like action-outcome learning or other forms of goal-directed behaviors will be affected similarly to what has been observed in reward-based circuits modified by social stress paradigms.

## Author Contributions

GH and DB wrote the manuscript.

## Conflict of Interest

The authors declare that the research was conducted in the absence of any commercial or financial relationships that could be construed as a potential conflict of interest.
